# Family Planning Counseling in Your Pocket: A Mobile Job Aid for Community Health Workers in Tanzania

**DOI:** 10.9745/GHSP-D-15-00393

**Published:** 2016-06-20

**Authors:** Smisha Agarwal, Christine Lasway, Kelly L’Engle, Rick Homan, Erica Layer, Steve Ollis, Rebecca Braun, Lucy Silas, Anna Mwakibete, Mustafa Kudrati

**Affiliations:** a FHI 360, Durham, NC, USA; b University of North Carolina, Gillings School of Global Public Health, Chapel Hill, NC, USA; c University of San Francisco, School of Nursing and Health Professions, San Francisco, CA, USA; d D-tree International, Dar es Salaam, Tanzania; e Pathfinder International, Dar es Salaam, Tanzania

## Abstract

To address low contraceptive use in Tanzania, a pilot intervention using a mobile job aid was developed to guide community health workers (CHWs) to deliver integrated counseling on family planning, HIV, and other sexually transmitted infections (STIs). In this article, we describe the process of developing the family planning algorithms and implementation of the mobile job aid, discuss how the job aid supported collection of real-time data for decision making, and present the cost of the overall system based on an evaluation of the pilot. The family planning algorithm was developed, beginning in June 2011, in partnership with the Tanzania Ministry of Health and Social Welfare based on a combination of evidence-based tools such as the Balanced Counseling Strategy Plus Toolkit. The pilot intervention and study was implemented with 25 CHWs in 3 wards in Ilala district in Dar es Salaam between January 2013 and July 2013. A total of 710 family planning users (455 continuing users and 255 new users) were registered and counseled using the mobile job aid over the 6-month intervention period. All users were screened for current pregnancy, questioned on partner support for contraceptive use, counseled on a range of contraceptives, and screened for HIV/STI risk. Most new and continuing family planning users chose pills and male condoms (59% and 73%, respectively). Pills and condoms were provided by the CHW at the community level. Referrals were made to the health facility for pregnancy confirmation, injectable contraceptives, long-acting reversible contraceptives and HIV/STI testing. Follow-up visits with clients were planned to confirm completion of the health facility referral. The financial cost of implementing this intervention with 25 CHWs and 3 supervisors are estimated to be US$26,000 for the first year. For subsequent years, the financial costs are estimated to be 73% lower at $7,100. Challenges such as limited client follow-up by CHWs and use of data by supervisors identified during the pilot are currently being addressed during the scale-up phase by developing accountability and incentive mechanisms for CHWs and dashboards for data access and use.

## Abstract (precis)

Using mobile job aids can help CHWs deliver integrated counseling on family planning and HIV/STI screening by following a step-by-step service delivery algorithm. Lessons learned during the pilot led to the development of additional features during scale-up to exploit the other major advantages that mHealth offers including:

- Better supervision of health workers and accountability for their performance
- Improved communication between supervisors and workers
- Access to real-time data and reports to support quality improvement

## BACKGROUND

Family planning use protects the health of women and their children by spacing births, preventing unwanted or high-risk pregnancies, reducing the need for abortions, and preventing mother-to-child transmission of HIV/AIDS, ultimately leading to a reduction in maternal and child deaths.^1^^,^^2^ One critical determinant of adoption and continuation of contraceptives is overall client satisfaction with family planning services.^3^ Therefore, provision of good-quality contraceptive services is vital to reducing unmet need for family planning. This includes broadening method choice and ensuring that any health concerns related to family planning are adequately addressed. Additionally, community-based family planning programs can bring quality contraceptive information and products to families in their communities, rather than requiring them to visit a health facility, thereby facilitating access to family planning services.^4^ Furthermore, evidence suggests that integration of family planning services with maternal health care and HIV/AIDS services is feasible and can result in overall improvements in contraceptive use as well as in antiretroviral therapy initiation in pregnancy, HIV testing, and quality of services.^5^

In Tanzania, current contraceptive use is low; 34.4% of women of reproductive age use any form of contraception, and only 27.4% use modern contraceptive methods.^6^ Further, over a quarter of women report unmet need for family planning.^6^ A review of family planning use in Tanzania identified widespread community misconceptions about contraceptives and poor confidence in the competence of community service providers as key factors limiting adoption.^2^

Poor community confidence in the competence of CHWs is one key factor limiting contraceptive adoption in Tanzania.

To address these service delivery gaps, FHI 360, Pathfinder International, and D-tree International (referred to as the “partnership”), with funding from the United States Agency for International Development (USAID), collaborated to develop a mobile phone job aid to assist community health workers (CHWs) in Tanzania with delivering family planning services. The impetus for this effort was a growing body of research showing that use of mobile phones to deliver health services by CHWs is feasible and well-received by the community and the health worker, and it can potentially result in improvements in adherence to service delivery protocols.^7^^–^^10^ The family planning mobile job aid program was planned to integrate with Pathfinder International’s successful community home-based care program,^11^ which was already using mobile job aids for case management of people living with HIV.

A mobile job aid was developed to assist CHWs in Tanzania with providing integrated family planning and HIV counseling and services.

Mobile phone applications may help CHWs adhere to service delivery protocols.

The partnership began initial development of the mobile job aid in June 2011. At that time, CHWs were using paper-based job aids and paper reporting forms, which posed significant challenges for service provision, data management, and reporting. The paper flip charts used for counseling women about contraceptive methods did not provide step-by-step guidance for the CHWs, and they were often cumbersome to carry. CHWs were required to record client data using multiple paper forms and to physically submit these to the health facility nurse, who would then physically submit the information to the district reproductive health coordinator. This led to considerable lag time between the point of collecting data from the client and reporting it at the district level, and potential for data loss. The mobile job aid was developed to address these challenges.

The purpose of this article is to describe our process for developing the family planning mobile job aid and to present data from a study evaluating the effectiveness of the pilot program in supporting collection of real-time data for decision making. Additionally, we present the cost of developing this system to facilitate an understanding of potential cost savings and efficiencies that might be possible through scale-up of such a system.

## COMPONENTS OF THE MOBILE JOB AID

The mobile job aid provides 3 main functions:

- **Decision-support tool:** An algorithm guides CHWs to effectively counsel, screen, and provide health facility referrals for pregnancy, sexually transmitted infections (STIs) including HIV, and family planning services.
- **Data collection and management tool:** Electronic forms help the CHW record routine data on services provided to the client, use of contraceptives by the client, and referrals to other health services. Information about each client is recorded at the point of care and then either sent through a general packet radio service (GPRS) linkage to a central database hosted at the CommcareHQ server (the platform used to develop the mobile job aid) or stored in the phone to be uploaded to the server when the CHW returns to the clinic. These data can be immediately accessed by the district-level health staff.
- **Short message service (SMS)-based management tool:** An SMS feature supports both the supervisors and the CHWs by issuing reports and reminders about the performance of the CHWs in the field. For example, the phone is designed to send SMS-based weekly status reports to CHW supervisors including the number of clients visited, number of new family planning users by contraceptive method, and number of referrals and completed follow-up visits made by CHWs. It is also designed to send SMS-based reminders to CHWs for followup visits to specific clients.

## PROCESS OF DEVELOPING THE MOBILE JOB AID

### Stakeholder Engagement

We developed the mobile job aid and implemented the study to evaluate its effectiveness in close collaboration with the Tanzania Ministry of Health and Social Welfare (MOHSW) at the national and district levels. For the evaluation of the tool, the team worked with the MOHSW to select the study sites and CHWs. Staff from the MOHSW were among the trainers who introduced the job aid to the CHWs and supervised the CHWs on a daily basis. Monthly meetings were held with CHWs and supervisors to assess progress and understand challenges. Finally, data were first shared with the MOHSW at all levels for their recommendation for next steps in implementation.

### Development of the Algorithm

The family planning mobile job aid was built using CommCare. CommCare uses JavaRosa, an open-source mobile and web platform designed for data collection. It was deployed on Nokia X2-02 phones, provided by the project to CHWs at the time of training. The algorithm for the mobile job aid is composed of 5 forms that link with each other depending on the choices the clients make:

- Registration form
- Service form for registered clients who are new to family planning
- Service form for registered clients who are continuing family planning users
- Follow-up forms for all clients
- Referral completion form

D-tree International programmed the initial algorithm on the CommCare platform and conducted various tests with the team to ensure that the phone application matched the paper-based version of the algorithm.

The algorithm is a combination of evidence-based tools, including the Balanced Counseling Strategy Plus Toolkit from the Population Council,^12^ the Decision-Making Tool for Family Planning Clients and Providers from the World Health Organization,^13^ and the pregnancy checklist for family planning clients from FHI 360.^14^ The partnership, together with the MOHSW, reviewed potential tools and agreed on criteria for tool selection. These criteria included the potential ease of use by CHWs, alignment with national family planning service protocols, and practicability as a mobile job aid.

**FIGURE 1:**
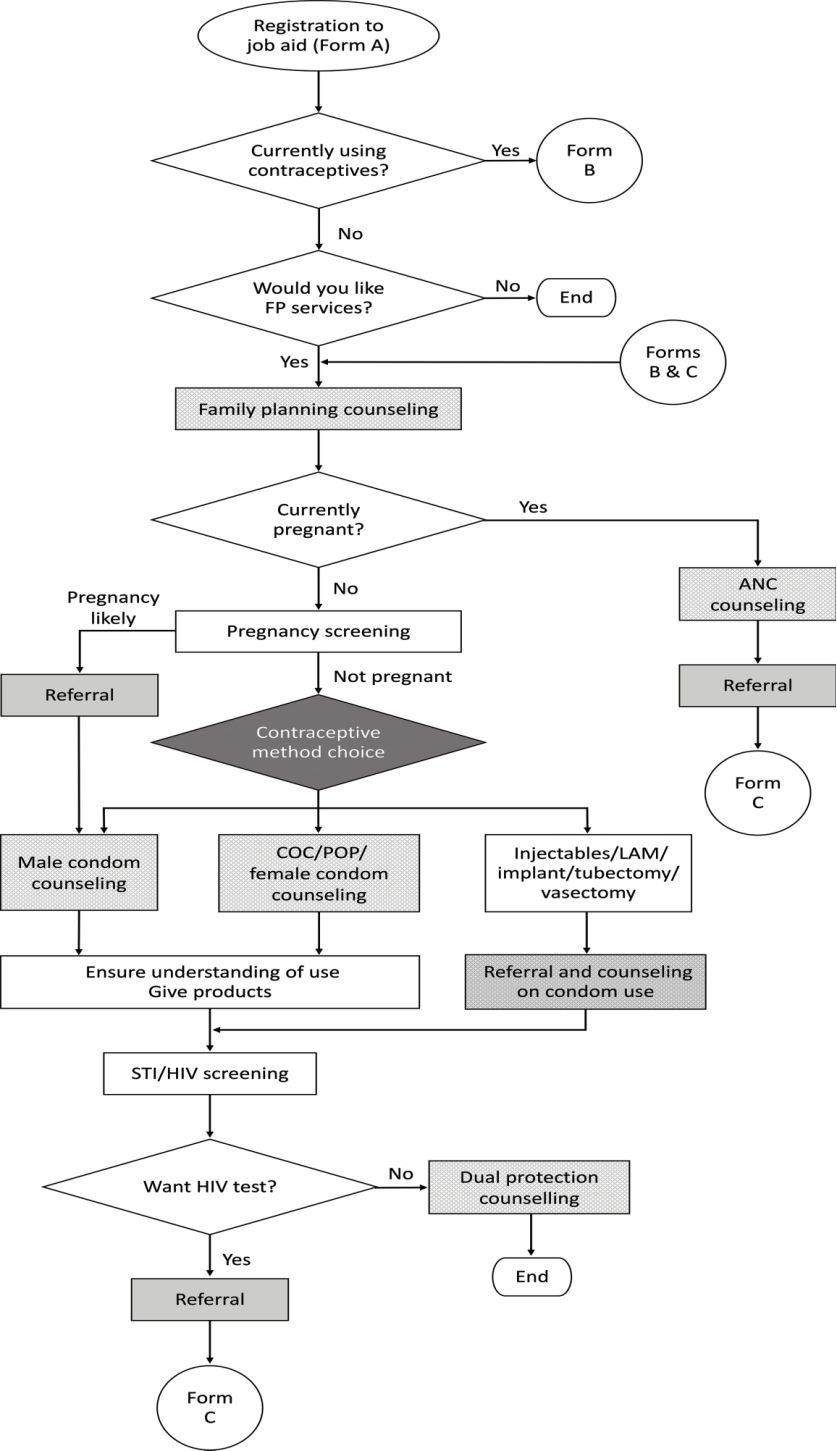
Family Planning Counseling Algorithm Abbreviations: ANC, antenatal care; COC, combined oral contraceptive; FP, family planning; LAM, lactational amenorrhea method; POP, progestin-only pill; STI, sexually transmitted infection.

**FIGURE 2:**
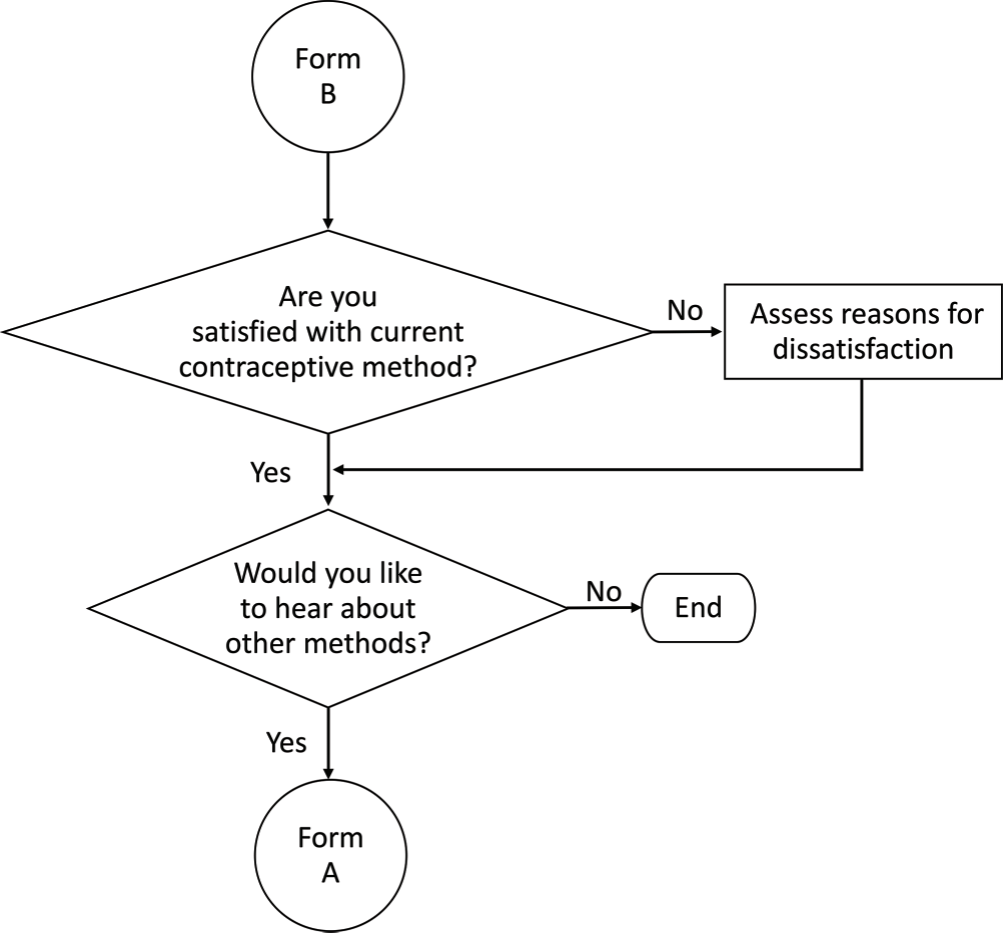
Follow-Up Algorithm to Assess Satisfaction With Current Contraceptive Choice

**FIGURE 3:**
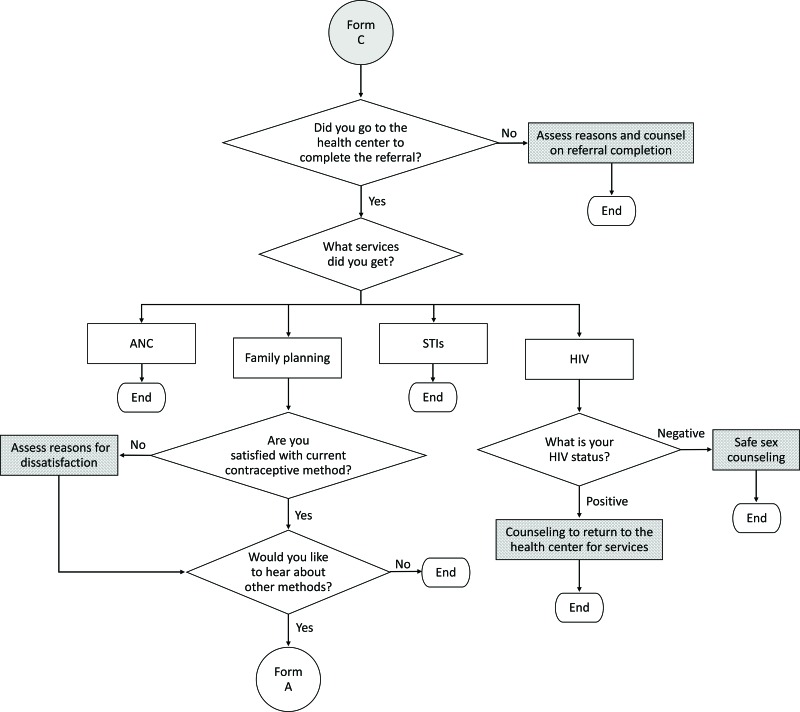
Referral Completion Algorithm for Clients Who Received a Health Facility Referral Abbreviations: ANC, antenatal care; STIs, sexually transmitted infections.

Figure 1 depicts the overall algorithm that guides the CHW through a series of steps: screening the client for pregnancy, counseling for a contraceptive method of choice and male condoms, providing contraceptives or facility referral for contraceptives that cannot be given at the community level, and conducting STI/HIV screening. If the client is already using contraceptives, the algorithm skips to the follow-up form in Figure 2. Finally, for clients that have been referred to the health facility, the CHW conducts a follow-up visit to assess whether the client completed the referral and requires any further services (Figure 3).

### Usability Testing and Refinement

The team worked with health facility supervisors to identify 6 highly motivated and well-performing CHWs from the Kinondoni municipal district in Dar es Salaam, Tanzania, to test the usability and functionality of the initial version of the tool for a period of 6 months, starting July 2012. This refinement period explored operational issues such as client reactions and the functionality of the tool. Through an iterative process involving use of the job aids by the CHWs, feedback, and field observations, the application was modified to better fit the needs of CHWs in the field.

## MOBILE JOB AID INTRODUCTION AND TRAINING

The district health management teams were introduced to the mobile job aid in an orientation session with the assistance of the MOHSW. All CHWs included in the pilot study received Nokia X2-02 mobile phones for the purpose of training and use during the study. CHWs and supervisors were trained over a period of 10 days, comprising 5 days of classroom training and 5 days of practical training in the field. Classroom training consisted of a family planning refresher training for 2 days, followed by 3 days of training using the mobile job aid. After completion of the training, the CHWs and supervisors started using the job aids at their sites. Support on the use of the tools was provided on a regular basis for 6 months, between January 2013 and July 2013.

## PROCESS EVALUATION OF THE MOBILE JOB AID

The results of the feasibility study, which included baseline and follow-up surveys and in-depth interviews with clients, CHWs, and facility supervisors, are presented in a separate manuscript.^15^ Here, we focus on data collected as part of routine electronic data collection during the process of service delivery.

### Site Selection

The use of the mobile job aid was implemented in 3 health facilities in 3 different wards of Ilala district in the Dar es Salaam region. The team and MOHSW purposively selected the implementation sites to represent sites where Pathfinder had established community-based care services for people living with HIV. Each health facility had 1 CHW supervisor and 5 to 13 CHWs. A total of 25 CHWs participated in the pilot study. This cohort of CHWs can be considered representative of the CHWs in Ilala district.

### Data Sources

Throughout the pilot period, the CHWs used the mobile job aids not only to provide counseling but also to enter data on the choices the clients made and the counseling messages that were provided to them. This system captured data on the number of new clients, average number of CHW visits per client, type of service sought, number of referrals completed, and other critical variables defined by the algorithm. In addition to this monitoring system, data on the incremental costs associated with the intervention were collected by retrospectively reviewing project documents.

## FINDINGS

### Background Characteristics of Family Planning Clients

Over the 6-month implementation period, 710 clients were registered and received family planning counseling using the mobile job aid: 455 clients (64%) who were already using some form of contraception (continuing users) and 255 (36%) who were not using any contraceptives (new users). Over a quarter of all users were male, and over 90% were married (Table 1). The majority (over 70%) of users were between ages 20 and 39 years.

**TABLE 1:** Demographic Characteristics, Contraceptive Choices, and HIV Testing of Continuing and New Family Planning Clients (N = 710)

	Continuing Users (n = 455) No. (%)	New Users (n = 255) No. (%)
Marital status		
Married	435 (95.6)	233 (91.4)
Not married	20 (4.4)	22 (8.6)
Sex		
Male	124 (27.3)	71 (27.8)
Female	331 (72.7)	184 (72.2)
Age, years		
15–19	37 (8.1)	26 (10.2)
20–29	169 (37.1)	106 (41.6)
30–39	182 (40.0)	79 (31.0)
≥40	67 (14.7)	44 (17.3)
Type of contraceptive method used^a^		
Male condoms	158 (34.7)	88 (34.5)
Pills (COCs or POPs)	173 (38.0)	61 (23.9)
Medium- or long-acting methods (DMPA injectables, implants, IUD)	57 (12.5)	54 (21.2)
Female condoms	60 (13.2)	17 (6.7)
Other (LAM, SDM, tubal ligation)	7 (1.5)	6 (2.4)
No method chosen	NA	29 (11.3)
Recently tested for HIV		
Yes	331 (72.8)	149 (58.4)
No	124 (27.2)	106 (41.6)

Abbreviations: COCs, combined oral contraceptives; DMPA, depot medroxyprogesterone acetate; IUD, intrauterine device; LAM, lactational amenorrhea method; POPs, progestin-only pills; SDM, Standard Days Method.

a For continuing users, type of method used at the time of the first visit by the CHW; for new users, the type of method selected after counseling by the CHW.

### Contraceptive Choices

#### Continuing Users

The 455 clients who were already using contraception received a total of 1,044 follow-up visits from the CHWs over a period of 6 months. At the time of the first visit by the CHW, nearly 35% of the continuing users were using condoms and 38% were using oral contraceptive pills (Table 1).

#### New Users

The 255 new clients received a total of 269 follow-up visits from the CHWs. During the visits, new clients were asked whether they would like family planning information. After receiving client consent to receive family planning information (n = 244), CHWs screened the clients for whether they were currently pregnant. The majority (n = 232) of clients stated they were planning to have a baby in the near future, and so these clients were educated on short-acting contraceptive methods. The remaining 12 clients were educated on long-acting contraceptives and permanent methods.

Clients were also asked whether they had support from their partner to use contraception. Of the 244 clients, 20 clients stated they did not have partner support. Based on their response, all clients were additionally counseled on methods that do or do not require cooperation by the partner. After this initial counseling, clients were asked whether they would like to choose a contraceptive method. Once clients chose a method, CHWs provided further counseling about their chosen method, including indications, contraindications, and possible side effects, if applicable. Even after making a choice, clients were asked if they would like to know about other methods, so information and counseling on all contraceptive methods was available to them.

The large majority of new family planning clients proceeded with making a contraceptive choice as shown in Table 1. Similar to continuing users, male condoms (35%) and pills (24%) were the most preferred contraceptive choice of new users, followed by medium-acting (injectables) or long-acting reversible contraceptives (LARCs) (21% combined). For clients who chose pills or condoms (male or female), the CHW provided the client with that method. Clients choosing injectables or LARCs were referred to the health facility.

Most continuing and new family planning clients chose to use male condoms or pills.

### HIV Counseling and Testing

All 710 clients received education, risk assessment, and pretest counseling for HIV and other STIs. The majority of both the continuing users (73%) and the new users (58%) said they had been recently tested for HIV. Those who said they had not been recently tested were assessed for HIV risk and counseled to get tested for HIV. Among those who had been recently tested and were willing to share their test results, 57% of the 276 continuing users and 54% of the 138 new users, tested HIV positive. The high percentage of individuals with HIV in the study is due to the fact that the mobile job aid intervention was piloted as part of Pathfinder’s community home-based care for people living with HIV/AIDS program. The clients were asked whether they were receiving HIV-related service at a health facility or at home, and the data were recorded. If clients were not receiving any care, they were registered for home-based HIV care services. If needed, clients were referred to the health facility for further testing services.

### Referral Services

Clients were referred to the health facility for services such as HIV/STI testing and contraceptive methods that could not be directly provided by the CHW at the community level. For example, 21% of new family planning users and 28% of continuing family planning users were referred for HIV testing/STI services. Referral completion rates and satisfaction with services received at the referral center were measured as reported by the client and entered into the mobile job aid. If the CHW did not follow-up with the client about the referral, these data were not collected. Of the clients who received referrals *and* follow-up by the CHWs within 6 months (n = 77), nearly 50% had completed their referrals.

Only about half of the CHW clients who received referrals to the health facility actually completed their referrals.

### Cost Assessment

Table 2 presents the costs associated with implementing the family planning mobile job aid with 25 CHWs and 3 supervisors across the 3 pilot health facilities. About 850 unique clients were served during the period of the pilot intervention. Costs for the deployment phase consisted of equipment purchases (e.g., mobile phones), adaptation of the paper-based job-aids to an electronic format, and training of the CHWs and supervisors on how to use the devices. The operational phase is focused on the monthly resources (primarily labor) and other supplies (primarily SMS fees and Internet access) required to keep the system operational. Costs of intervention design, stakeholder engagement, and content development were not included in this analysis. The results presented here reflect the actual financial costs incurred to support the deployment and operation of the mobile job aid intervention.

**TABLE 2:** Total Financial Costs Associated With Implementing the Family Planning Mobile Job Aid in 3 Pilot Facilities

Phase	Total Cost (US$)
**Deployment**	
Sourcing equipment	1,875
Adaptation of the paper-based job aids to electronic format	13,500
Training of 25 CHWs and 3 supervisors	3,522
**Operational (annual costs)**	
Service provision, reporting, and supervision	1,014
IT support and troubleshooting	3,600
SMS & Internet access fees, replacement of handsets	2,494
**Total resource requirement for first year** (deployment and operational phases)	**26,005**
**Total resource requirement for subsequent years** (operational phase only)	**7,107**

Abbreviations: CHWs, community health workers; SMS, short message service.

The total value of resources required to implement the mobile job aid is estimated to be US$26,005 in the first year (Table 2). Over 50% of the financial costs in the first year correspond to the cost of adapting the existing paper-based job aids and reporting forms to an electronic version compatible with the handheld device. The training of CHWs and supervisors is estimated to require 14% of the resources in the first year. Financial costs in subsequent years ($7,107) are estimated to be 73% lower than in the initial year ($26,005), and approximately 51% of these costs are for IT support and field troubleshooting. It should be noted that these costs reflect program costs under trial settings. Larger-scale implementation of a similar project would require additional management and infrastructure support, which would add to the overall costs.

## DISCUSSION

Our experience from this pilot study suggests that the use of a mobile-based integrated counseling algorithm is feasible and can potentially result in improvements in community-based family planning service delivery. As part of this process, we were able to bring together diverse stakeholders to identify best practices and to develop a systematic algorithm that combined counseling for reproductive health, family planning, and STIs/HIV. The usability testing and refinement phase ensured that the job aid was well-received by the CHWs and the clients.

The mobile tool guides CHWs through the algorithm step-by-step and prevents skipping over critical questions and information during counseling. Additionally, it allows for immediate collection and digitization of the data, and it facilitates easier data use across different levels of the health system. Preliminary research on the use of such tools by CHWs suggests that real-time data collection can influence CHW motivation and improve accountability.^16^ As depicted in this paper, data from routine service delivery can be used to understand who the clients are, their contraceptive preferences, and the services for which they are being counseled and referred. The use of routine monitoring data can also help identify areas where the program can be improved.

### Lessons Learned

While the counseling algorithm can support the systematic delivery of information about a wide range of contraceptives, actual client use of contraceptives is still influenced by the contraceptive knowledge of the CHWs, the client’s readiness to start using contraception, and the availability of contraceptives methods. Even though LARCs and injectables are more prevalent in the current method mix in Tanzania than previously,^17^ most people in our study received either male condoms or oral contraceptive pills. It is possible that CHWs counsel more extensively on methods such as pills and condoms because they are more familiar with these methods. It is also plausible that clients chose pills and condoms over other methods because they were immediately available from the CHW and did not require a health facility visit. In contrast, to obtain injectables, LARCs, or permanent methods, clients had to go to the health facility. These data emphasize the need to continue training CHWs on balanced and comprehensive counseling techniques. The mobile job aid should serve as an adjunct—not a substitute—for continued investments in human resources and health systems. Additionally, systems to encourage CHWs to follow-up on clients more systematically are critical. Our study suggested that less than 50% of the clients who were referred to the health facility were followed-up by the CHWs to confirm referral completion.

Mobile phone applications should serve as an adjunct—not a substitute—for continued investments in human resources and health systems.

**Figure:**
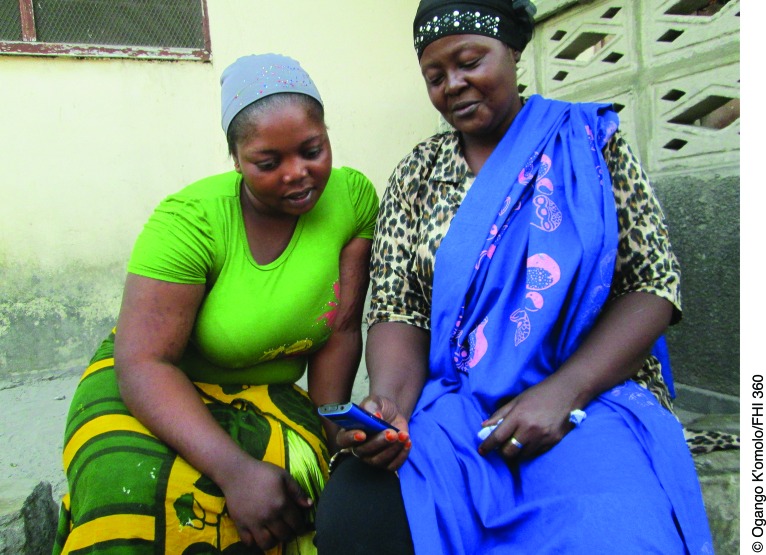
A community health worker in Kipawa, Dar es Salaam, uses a mobile phone job aid to provide integrated family planning and HIV/STI counseling to a client.

Integrated delivery of family planning with other health services is identified as a key strategic goal of the Tanzania MOHSW.^17^ In practice, integration requires the development of comprehensive packages of multiple health services, making the job aid more complex. For purposes of implementation by CHWs, it is critical to balance the content of such a package to what is critical to service delivery to avoid making it time consuming and unwieldly. As such, efforts are ongoing to further refine the family planning algorithm presented here.

While the collection of routine service provision data makes data available to districts, it does not mean that the data are actually used at the district level. The SMS component of our intervention was intended to support the district staff in decision making. However, no additional accountability mechanisms were developed to facilitate or monitor data use at that level. Additionally, our study generated a considerable amount of routine data for each client who was registered to the system. However, use of these data for meaningful analytics was challenging due to multiple skip patterns in the algorithm and some variability in storing and recording the data. A priori understanding of what data are critical to decision making and use of data dashboards can help to alleviate some of these challenges.

Finally, data on completion of referrals by clients were self-reported and recorded only if the CHW returned to the client to follow-up. The CHWs have mobile connectivity but these data are not linked to the health facilities. This limits the assessment of referral completion. Strengthening facility-level health information systems (HIS) and integration of the job aid with the HIS can yield more accurate data on referral completion and other critical outcomes when the client actually visits the health facility.

### The Way Forward and Scale-Up

Since this initial pilot project was launched, Pathfinder and D-tree have scaled up this work with 250 CHWs in northwest Tanzania. Several challenges identified during the pilot stage, such as poor follow-up of clients by CHWs and limited use of data at all levels of the health system, are being addressed. Modifications have improved systems for CHW motivation, supervision, and access to data for decision making. To motivate CHWs and hold them accountable for completing their work, a pay-for-performance system was implemented that provides additional mobile phone minutes to CHW stipends for meeting targets for registering a minimum number of new clients each month and completing 75% or more of scheduled follow-up visits. A custom supervisory application was developed for supervisors at health facilities that allows them to review CHW performance in real time, communicate about family planning outreach services and method stock, and view aggregate government reports. In contrast to the SMS management tool developed for the original job aid, which provided static weekly messages, this mobile app provided real-time data to supervisors and allowed them to connect with CHWs in the field in real time, strengthening the relationship and providing both CHWs and supervisors with more dynamic access to information and communication. In addition, CHWs and supervisors are part of a closed user group, which allows users to make free phone calls to other members of the group. This has improved communication and supervision among CHWs, who are often located in remote villages far from the nearest health facility. A “citizen report card” was also added to the mobile application, which assesses client experiences at health facilities after receiving a referral. Data are used to discuss the quality of care at facilities and to engage in constructive dialogue with individuals throughout the health system to develop strategies for quality improvement. Finally, program dashboards have been developed that provide interactive charts and tables summarizing key data from the programmatic level down to patient-level data. This supports program managers and supervisors at the district and regional levels to view data and make programmatic decisions in real time.

The mobile phone initiative has been scaled up from 25 CHWs to 250 CHWs in northwest Tanzania.

## CONCLUSION

The use of mobile job aids for delivery of integrated family planning services holds great promise. However, in order to scale effective programs, a critical appraisal and open discussion of the challenges and solutions is necessary.

## Notes

**Peer Reviewed**
